# Automated three-dimensional computed tomography analysis for surgical decisions in congenital nasal pyriform aperture stenosis

**DOI:** 10.1007/s00247-025-06282-7

**Published:** 2025-06-24

**Authors:** Talia Yeshua, Yoav Ben-Haim, Yehuda Schwarz, Pierre Attal, Eliyahu Nezri, Shlomo Grinzaig, Eliel Ben-David

**Affiliations:** 1 Jerusalem College of Technology, Jerusalem, Israel; 2 Hebrew University of Jerusalem, Jerusalem, Israel; 3 Shaare Zedek Medical Center, Jerusalem, Beyth St 12, 9103102 Israel

**Keywords:** Airway management, Computed tomography, Computer-assisted decision-making, Congenital nasal pyriform aperture stenosis, Image interpretation, Computer-assisted, Three-dimensional imaging

## Abstract

**Background:**

Congenital nasal pyriform aperture stenosis is a rare neonatal condition that causes respiratory distress and potentially requires surgery. Current diagnosis relies on clinical assessment and manual computed tomography (CT) measurements of the pyriform aperture width, which may not fully capture obstruction severity.

**Objective:**

To evaluate the severity of pyriform aperture stenosis using automatic three-dimensional (3-D) analysis and identify parameters discriminating between conservative and surgical cases.

**Materials and methods:**

This retrospective study analyzed CT scans of neonatal airways using a novel automated 3-D segmentation algorithm. We collected 22 CT scans (2010–2022) of newborns aged 0–35 days: 12 controls, four moderate cases treated conservatively, and six severe cases requiring surgery. The algorithm measured pyriform aperture width, nasal volumes, surface area, and cross-sectional areas.

**Results:**

The algorithm achieved high accuracy (Dice coefficient, 0.961 ± 0.005) and aligned well with manual measurements of the pyriform aperture (average difference, -0.05 ± 0.77 mm, -0.7 ± 9.1%). All cases with stenosis showed anterior narrowing, while only severe cases exhibited stenosis along the entire mid-nasal cavity. Mid-nasal cavity volume, surface area, and cross-sectional areas at 50% and 75% of the mid-nasal cavity emerged as potential surgical predictors, with cross-sectional area at 75% being the most discriminating (moderate, 68.6 ± 7.5 mm^2^; severe, 33.5 ± 13.7 mm^2^; *P*<0.01).

**Conclusion:**

Automated 3-D CT analysis quantifies pyriform aperture stenosis severity by measuring nasal airway dimensions. The study suggests objective parameters that may assist in surgical decisions and highlights the importance of considering the entire 3-D nasal cavity when planning surgical interventions. A multi-center study with a larger cohort is recommended to validate these findings.

**Graphical Abstract:**

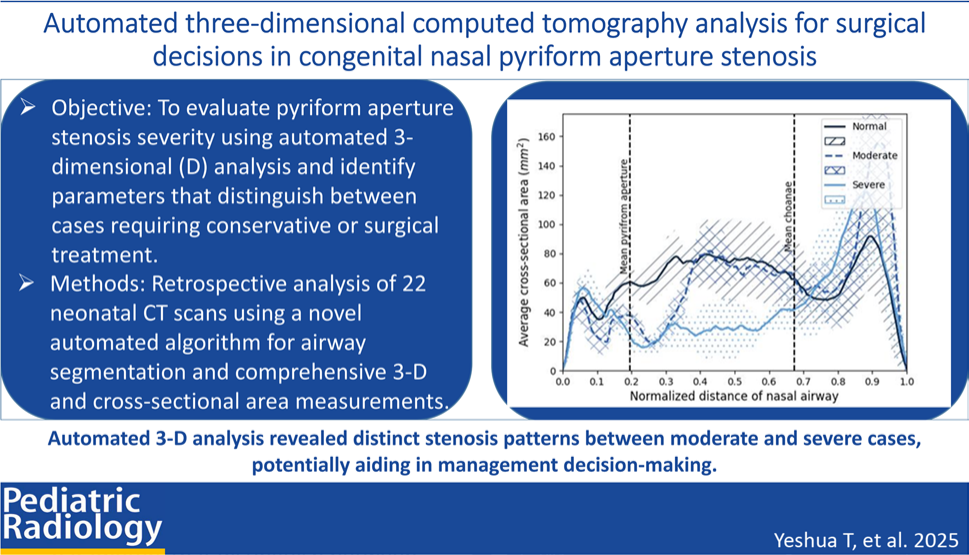

**Supplementary Information:**

The online version contains supplementary material available at 10.1007/s00247-025-06282-7.

## Introduction

Congenital nasal pyriform aperture stenosis is a rare condition characterized by bony overgrowth of the maxilla, resulting in a narrowing of the nasal airways in neonates [1–3]. This obstruction can lead to significant respiratory difficulties, posing potential risks to the overall health and development of infants [4, 5]. Early detection and accurate diagnosis of pyriform aperture stenosis severity are crucial to initiate appropriate medical interventions, including surgery when necessary [6, 7].

Currently, diagnosis of pyriform aperture stenosis relies primarily on clinical evaluation and computed tomography (CT) imaging with manual measurements of the pyriform aperture width [5, 6]. Pyriform aperture stenosis is typically diagnosed when the pyriform aperture width is narrower than 11 mm [2, 6]. Several researchers have proposed pyriform aperture width thresholds (5.7 mm [4], 6 mm [8]) as predictors of the need for surgical intervention in pyriform aperture stenosis. However, other researchers have questioned this approach and showed that the manual measurement of the pyriform aperture width alone does not fully capture obstruction severity or provide sufficient quantitative information to determine appropriate treatment [7, 9, 10].

Several studies [11–13] measured two-dimensional (D) distances between the bones at several points along the nasal cavity and found that the stenosis also occurs in posterior segments of the nasal cavity. Therefore, they suggested that focusing solely on the pyriform aperture width is insufficient to characterize the obstruction and determine the appropriate surgical method [11–13]. Nevertheless, they did not establish any objective metrics to assist in formulating surgical decisions. In addition, to the best of our knowledge, no extensive study has described the effect of obstruction on the entire 3-D nasal airway in pyriform aperture stenosis cases or established standard metrics for healthy newborns despite the potential for extracting 3-D nasal airway geometry from CT scans [14, 15]. This 3-D data could offer deeper insights into nasal obstruction and enable more informed surgical decisions and planning [11, 15–17].

This study aims to address these gaps by developing an algorithm to automatically segment nasal airways in neonates using CT images, diagnose pyriform aperture stenosis by measuring pyriform aperture width, and provide objective quantitative volumetric and cross-sectional information about the entire nasal airway dimensions. Our primary goal is to explore the impact of pyriform aperture stenosis on 3-D airway dimensions and investigate their potential as objective indicators for surgical decision-making and planning in neonates with pyriform aperture stenosis.

## Materials and methods

This retrospective study was designed to evaluate nasal airways in pyriform aperture stenosis using CT scans. The research plan consisted of three main components: (1) development of an automated algorithm for nasal airway segmentation and measurement, (2) validation of the algorithm accuracy against expert assessment, and (3) comparison of nasal dimensions between normal controls and stenosis cases requiring either conservative or surgical treatment. The study was conducted with ethics approval from our institution (No. 0305–21).

### Dataset

**Fig. 1:**
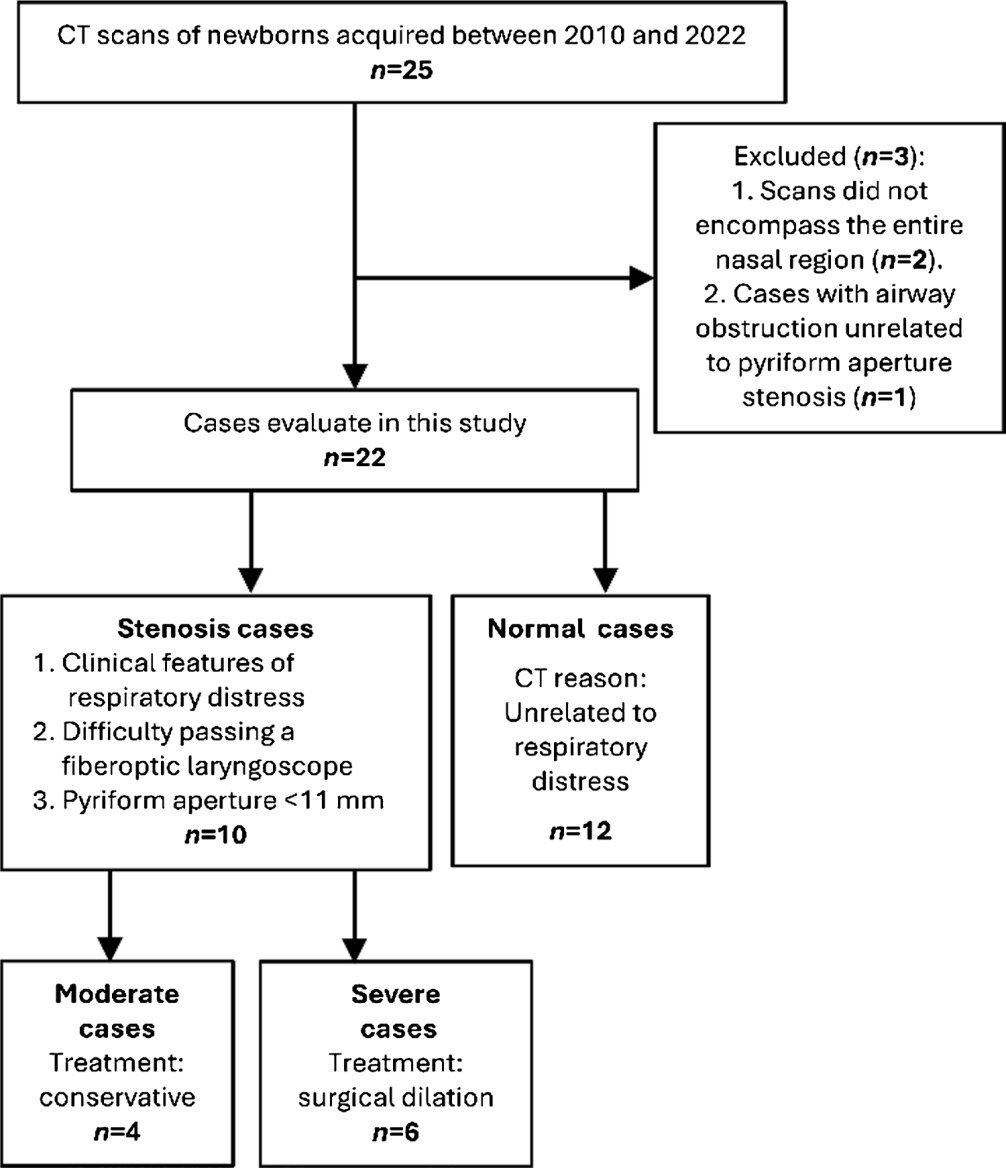
Patient selection flowchart for congenital nasal pyriform aperture stenosis study. *CT*, computed tomography

The dataset comprises 22 anonymized CT scans of newborns acquired at our institution between 2010 and 2022, as shown in the patient flow diagram (Fig. 1). Inclusion criteria for both groups were CT scans of neonates up to 35 days of age. Given the scarcity of neonatal CT scans due to radiation concerns, we extended the traditional neonatal period (28 days) to include cases up to 35 days, as diagnostic imaging of pyriform aperture stenosis was still being performed during this period. The control group included newborns with normal nasal airways who underwent CT scans for indications unrelated to respiratory distress. The inclusion criteria for the study cases were (1) clinical symptoms such as tachypnea, apnea episodes, nasal congestion, and feeding difficulties; (2) difficulty passing a fiberoptic laryngoscope past the anterior part of the nose; and (3) CT scan confirmation of a pyriform aperture width less than 11 mm. Exclusion criteria for the database were (1) CT scans that did not encompass the entire nasal region and (2) cases with airway obstruction unrelated to pyriform aperture stenosis. Patients were selected consecutively based on these criteria.

Conservative treatment was initially preferred for all pyriform aperture stenosis cases unless it failed or was contraindicated due to severe respiratory distress. Based on treatment response, stenosis cases (*n*=10) were categorized into two subgroups: “moderate” (*n*=4, managed successfully with conservative treatment) and “severe” (*n*=6, requiring surgical nasal airway dilation). The control group consisted of 12 cases. There were no significant differences in age or weight between the groups (Table 1). The pyriform aperture width of all cases was measured independently by a pediatric otolaryngologist (Y.S.) and a radiologist (E.B.D.), with their measurements showing agreement. Both physicians have over 17 years of experience in their respective fields.

**Table 1:** Demographic and clinical characteristics of normal, moderate, and severe cases

Characteristics	Normal cases (*n*=12)	Moderate cases (*n*=4)	Severe cases (*n*=6)
Sex (female:male)	7:5	2:2	1:5
Gestational age (weeks)	39.7 ± 0.8	39.9 ± 1.5	39.9 ± 1.9
Weight (kg)	3.12 ± 0.48	3.35 ± 0.27	3.15 ± 0.63
Age at CT (days)	10.1 ± 12.4	7.5 ± 4.4	12.8 ± 11.7
Manual measurements of pyriform aperture width (mm)	11.46 ± 1.09	4.98 ± 0.63	4.67 ± 1.28
Single median central incisor	N/A	1/4 (25%)	2/6 (33.3%)

Values are presented as mean ± standard deviation or count (percentage) as appropriate

*CT* computed tomography, *N/A* not applicable

**Fig. 2:**
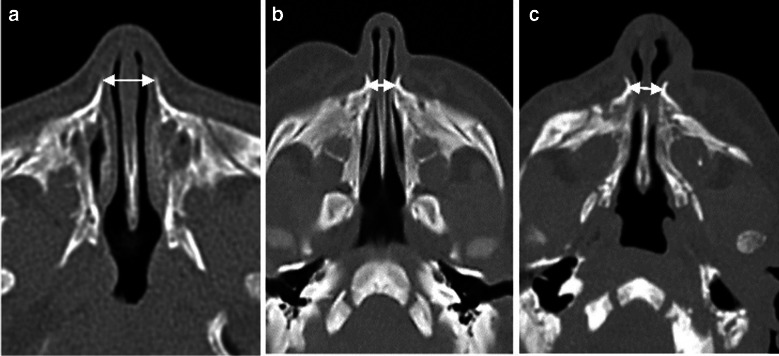
Axial computed tomography (CT) slices (without contrast) showing three examples of manual measurements of the pyriform aperture width using a bidirectional arrow. **a** A 4-day-old girl with normal pyriform aperture width (11 mm). **b** A 4-day-old girl with congenital nasal pyriform aperture stenosis treated conservatively (pyriform aperture width = 4.7 mm). **c** A 2-day-old boy with pyriform aperture stenosis who underwent surgery (pyriform aperture width = 6.7 mm)

Importantly, treatment decisions were based on clinical presentation rather than pyriform aperture width alone. Some cases with narrow pyriform aperture width were managed conservatively when symptoms were mild, while others required surgery despite larger pyriform aperture measurements due to severe respiratory symptoms, as demonstrated in Fig. 2. Detailed background data for each case is provided in Supplementary Material 1.

### Computed tomography scan details

The CT scans were sourced from four different Siemens CT SOMATOM models: Force, Definition AS +, Definition Flash, and Drive (Siemens Healthineers, Erlangen, Germany). The X-ray tube voltage typically operated at 70 kV or 100 kV, with current ranging from 40 to 190 mA. The CT dose index ranged from 1.5 mGy to 11.4 mGy. Computed tomography imaging consisted of a variable number of axial slices, ranging from 80 to 235 slices. These slices encompassed the region from the skull to the mouth and, in some cases, extended to the shoulders. The slice thickness was 1 mm, with an overlap ranging from 0.33 mm to 0.75 mm. Pixel size ranged between 0.2 mm and 0.4 mm, with a minimum image dimension of 512 × 512 pixels*.*

### Automatic algorithm

An algorithm was developed to automatically identify pyriform aperture stenosis and assess its severity using Python version 3.8 and above. The algorithm processes CT scans by first identifying all relevant nasal airways from the nostrils to the nasopharynx, up to the level of the soft palate. It then measures the pyriform aperture width and the nasal airway dimensions. The algorithm is fully automatic, aside from an optional manual reorientation step to correct head tilt via a simple graphical user interface (GUI). Segmentation completes in approximately 15 s on a standard desktop computer (Intel i7, 16GB random access memory (RAM)). The segmentation pipeline includes preprocessing, airway isolation, global and local thresholding, and post-processing for anatomical filtering. Core steps are described below. Full technical details, including parameter settings and implementation strategies, are available in Supplementary Material 2. A schematic workflow is also provided in Supplementary Material 2, and full code is available at https://github.com/yohod/NeonateNasalAirwayEvaluator.

**Fig. 3:**
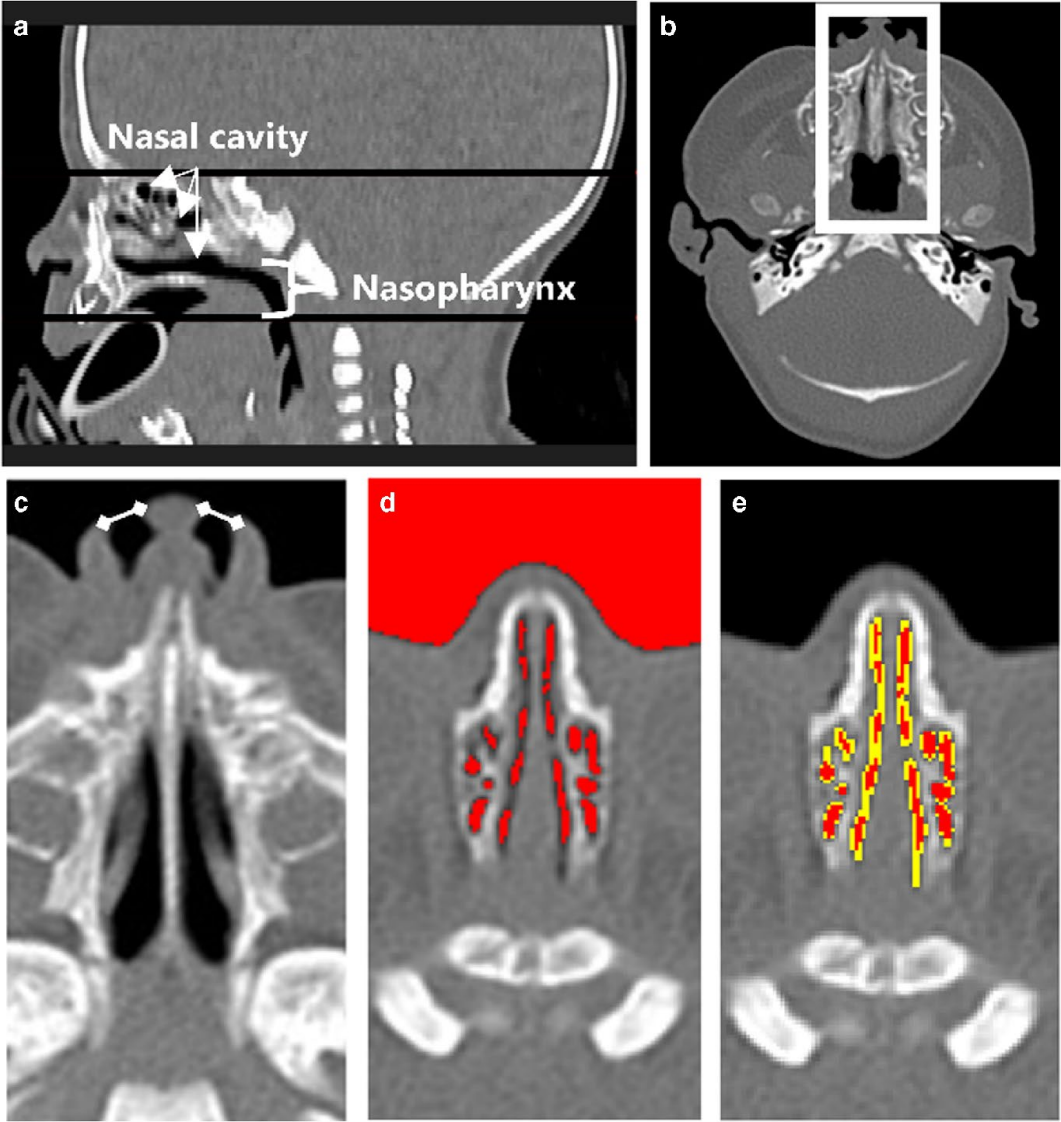
Automatic nasal airway segmentation process in an 8-day-old boy (computed tomography (CT) scan without contrast). **a**, **b** Selection of the volume of interest (VOI). **a** Reconstructed sagittal view showing the axial slices (*black lines*) that define the upper and lower boundaries of the nasal cavity and the nasopharynx, automatically selected by the algorithm. **b** Axial slice showing automatic cropping of the nasal airway region; a *white box* indicates the region of interest. **c**–**e** Nasal airway segmentation in axial view. **c** Nostril borders are defined by *straight lines* (*white lines*). **d** Initial segmentation using a global threshold of − 400 Hounsfield units (*red*, algorithm output) includes surrounding air, while narrow regions remain undetected. **e** Final segmentation after applying local adaptive thresholding and 3-connectivity filtering to include narrow regions (*yellow* indicates added areas, *red* indicates the initial segmentation; both are outputs of the automatic algorithm). The surrounding air was automatically removed

As shown in Fig. 3, the identification of the nasal airway starts by selecting the volume of interest (VOI), focusing on axial slices of the nasal airway extending to the nasopharynx. Within each slice, the relative region of interest (ROI) is designated. Lateral cropping is applied to retain only the central third of the head width, which consistently captures the nasal passage while excluding non-relevant head areas. Next, the algorithm defines the borders of the nostrils using straight lines, isolating the nasal cavities from external air. These lines are derived per slice by detecting the nostril contour and identifying two lateral boundary points; a virtual closure line is drawn between them. All voxels anterior to this line are excluded.

**Fig. 4:**
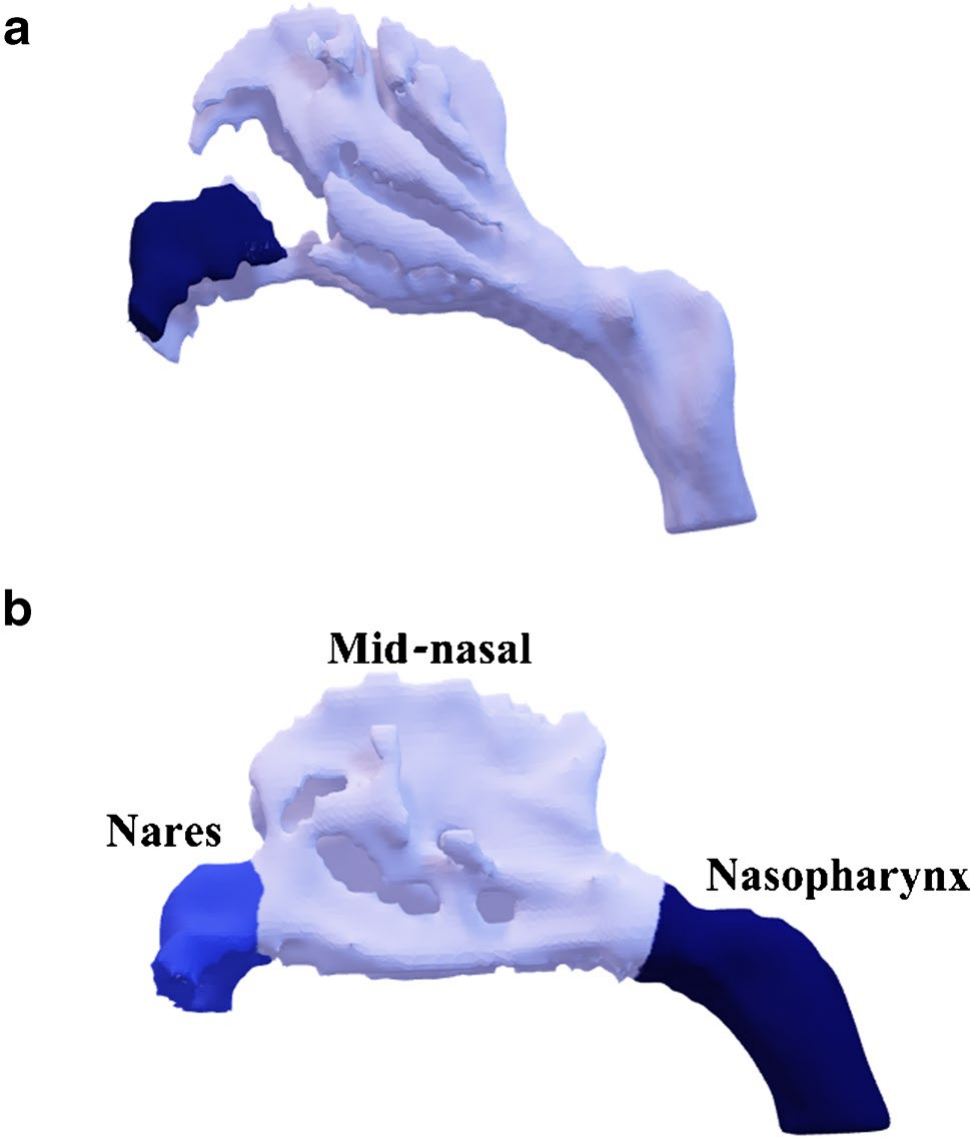
Three-dimensional (D) models of the nasal airway in two newborns. **a** A 5-day-old girl with congenital pyriform aperture stenosis, demonstrating complete obstruction of one nostril. The 3-D model highlights connected airway regions in *light blue* and anatomically isolated regions in *dark blue*. In this case, the isolated nostril (*dark blue*) reflects a true lack of continuity with the main nasal airway, corresponding to complete obstruction of one side. This color distinction allows for intuitive identification of fully blocked regions. **b** A 6-day-old boy with no complete obstruction. Regional airway volumes are illustrated: nares (*blue*), mid-nasal region (*light blue*), and nasopharynx (*dark blue*)

Following this, an initial airway segmentation is performed by identifying relatively low Hounsfield unit (HU) regions using a global threshold of − 400 HU. Small objects (smaller than 2 ${\text{mm}}^{3}$) and irrelevant regions are eliminated, such as surrounding external air and oropharyngeal cavities. Subsequently, the algorithm expands initial regions selectively to encompass narrow airway areas. This is done using adaptive local thresholding: voxels with HU between − 400 and − 125 are evaluated. A voxel is included if its value is below the local mean (computed over a 3 × 3 neighborhood excluding air voxels below − 400 HU) and if it is 3-D connected to the initial − 400 HU mask. This process improves sensitivity to low-contrast, narrow airways typical in neonatal scans. Finally, only airway regions that are 3-D connected to the central nasal passage are retained, revealing areas lacking anatomical continuity (Fig. 4).

For measuring segmented regions in the nasal airway, the algorithm automatically detects the coronal slices of the pyriform aperture and the choanae. The region situated between the pyriform aperture and the choanae is recognized as the primary site for most congenital nasal obstructions and commonly exhibits narrowing in pyriform aperture stenosis cases [11–13]. The coronal slice of the pyriform aperture was defined by scanning anterior-to-posterior coronal slices, starting at the nasal tip. The pyriform aperture was identified as the most anterior slice where a bone could be segmented using a + 200 HU threshold at the axial level of the nasal tip, which corresponds to the mid-to-inferior region of the pyriform aperture bones. This relatively low threshold allows the detection of under-ossified structures in neonates [18]. The coronal slice of the choanae was defined by scanning posterior-to-anterior coronal slices and measuring the width of the main connected airway cavity. The choanae were identified as the slice where this width decreased by ≥ 1.5 × compared to the previous slice, indicating a division of the airway into two separate nasal passages and marking the transition to the nasopharynx [19].

Various dimensions of the nasal airway are then measured, including total volume $\left({\text{cm}}^{3}\right)$, and segmented volumes within specific regions, namely, the nares region extending from the nostril to the pyriform aperture, the mid-nasal region situated between the pyriform aperture and the choanae, and the nasopharynx region spanning from the choanae to the terminus of the nasopharynx (Fig. 4). Surface area $\left({\text{cm}}^{2}\right)$ and the cross-sectional area along the canal $\left({\text{mm}}^{2}\right)$ are also quantified.

Finally, the algorithm measures the pyriform aperture width to evaluate whether the minimum width in the lower portion of the pyriform aperture is less than 11 mm, which raises suspicion of pyriform aperture stenosis [6]. The main parts of this step are displayed in Supplementary Material 2.

### Statistical analysis

The validation process [14, 20, 21] for the automated segmentation included an assessment by an expert radiologist (E.B.D.) and manual segmentation of four cases (two per group). Sensitivity [22] and Dice coefficient [23] then confirm the accuracy and reliability of the automated segmentation relative to the manual segmentation. The automated measurements of the pyriform aperture width were compared to manual measurements for all cases, to assess the performance of the algorithm in automatically measuring the pyriform aperture width.

To discern factors indicative of the degree of obstruction, a comprehensive comparative analysis of nasal dimensions was carried out. The statistical evaluation encompassed key parameters, including pyriform aperture width, total volume, regional volumes, and surface area. Furthermore, a graphical analysis of the combined cross-sectional area of both nasal cavities along the airway was generated. For each case, the *x*-axis of the graph was rescaled to represent the percentage of cavity length from the nostrils (0%) to the posterior nasopharyngeal wall (100%). The average cross-sectional area and corresponding standard deviation were calculated for each group and displayed graphically. In addition, the average cross-sectional area was compared at five locations spaced at 25% intervals along the nasal cavity. At each location, averaging was performed within a ± 5% range from the specified point within the mid-nasal cavity region, extending from the pyriform aperture (0%) to the choanae (100%).

All statistical analyses were performed using Pingouin (version 0.5.3), an open-source Python package for statistical analysis (University of California, Berkeley, CA) [24]. Considering the specific conditions of this study, the Mann–Whitney *U* test was selected, as it is well-suited for small sample sizes and cases where a normal distribution assumption is not applicable [25].

## Results

### Algorithm performance and measurements

The algorithm successfully processed all cases and achieved a sensitivity of 0.977 ± 0.003 and a Dice coefficient of 0.961 ± 0.005. Automatic and manual measurements of the pyriform aperture width showed good agreement (Supplementary Material 1) with an average difference of −0.05 ± 0.77 mm (−0.7 ± 9.1%).

### Nasal dimensions

The average nasal dimensions and corresponding *P*-values are consolidated in Table 2 (precise measurement details are in Supplementary Material 1). Notably, *P*_a_ and *P*_b_ are the *P*-values that denote the ability to differentiate between the normal group and each of the stenosis subgroups, moderate or severe, respectively. These values signify the diagnostic efficacy in distinguishing between healthy cases and those with stenosis. On the other hand, *P*_c_ assesses the index capability to signify obstruction severity within the moderate subgroups compared to the severe subgroup.

**Table 2:** Comparison of automatically measured nasal airway dimensions in normal, moderate, and severe cases

Nasal airway dimensions	Normal	Moderate	Severe
Pyriform aperture width (mm)	$11.8\pm 0.8$	$4.5\pm 0.9$ (***P***_**a**_**<0.01**)	$4.4\pm 1.4$ (***P***_**b**_**=0.01**, *P*_c_=0.30)
Nares volume (cm^3^)	$0.41\pm 0.10$	$0.36\pm 0.11$ (*P*_a_=0.20)	$0.42\pm 0.09$ (*P*_b_=0.54, *P*_c_=0.82)
Mid-nasal volume (cm^3^)	$2.03\pm 0.48$	$1.45\pm 0.18$ (***P***_**a**_**=0.01**)	$0.79\pm 0.40$ (***P***_**b**_**<0.01, *P***_**c**_**=0.03**)
Nasopharynx volume (cm^3^)	$1.04\pm 0.31$	$1.59\pm 0.80$ (*P*_a_=0.15)	$1.40\pm 0.53$ (*P*_b_=0.07, *P*_c_=0.62)
Total volume (cm^3^)	$3.48\pm 0.58$	$3.40\pm 0.79$ (*P*_a_=0.43)	$2.62\pm 0.87$ (***P***_**b**_**=0.02**, *P*_c_=0.13)
Surface area (cm^2^)	$47.5\pm 7.8$	$48.6\pm 4.9$ (*P*_a_=0.70)	$31.3\pm 9.5$ (***P***_**b**_**<0.01, *P***_**c**_**=0.02**)

Values are presented as mean ± standard deviation. *P*-values below 0.05 are shown in bold to indicate statistical significance

Reviewing the results presented in Table 2 confirms that the pyriform aperture width effectively diagnosed pyriform aperture stenosis (*P*_a_, *P*_b_<0.01) but lacked reliability in indicating the severity of the obstruction or the necessity for surgery (*P*_c_=0.30). Moreover, the volume and surface area measurements do not necessarily differentiate between normal and moderate cases. However, in severe cases, these parameters are smaller (*P*_b_=0.02, *P*_b_<0.01, respectively). Notably, the surface area even differentiated the severe from moderate cases (*P*_c_=0.02). This may reflect anatomical complexity relevant to surgical planning. Furthermore, mid-nasal volume is the sole index that distinguishes between normal and stenosis cases (*P*_a_=0.01, *P*_b_<0.01), and between moderate and severe obstructions (*P*_c_ = 0.03). The average nasopharynx volume in the normal group was about one-third smaller than in the stenosis subgroups. However, this difference was not statistically significant.

### Nasal cross-sectional area analysis

**Fig. 5:**
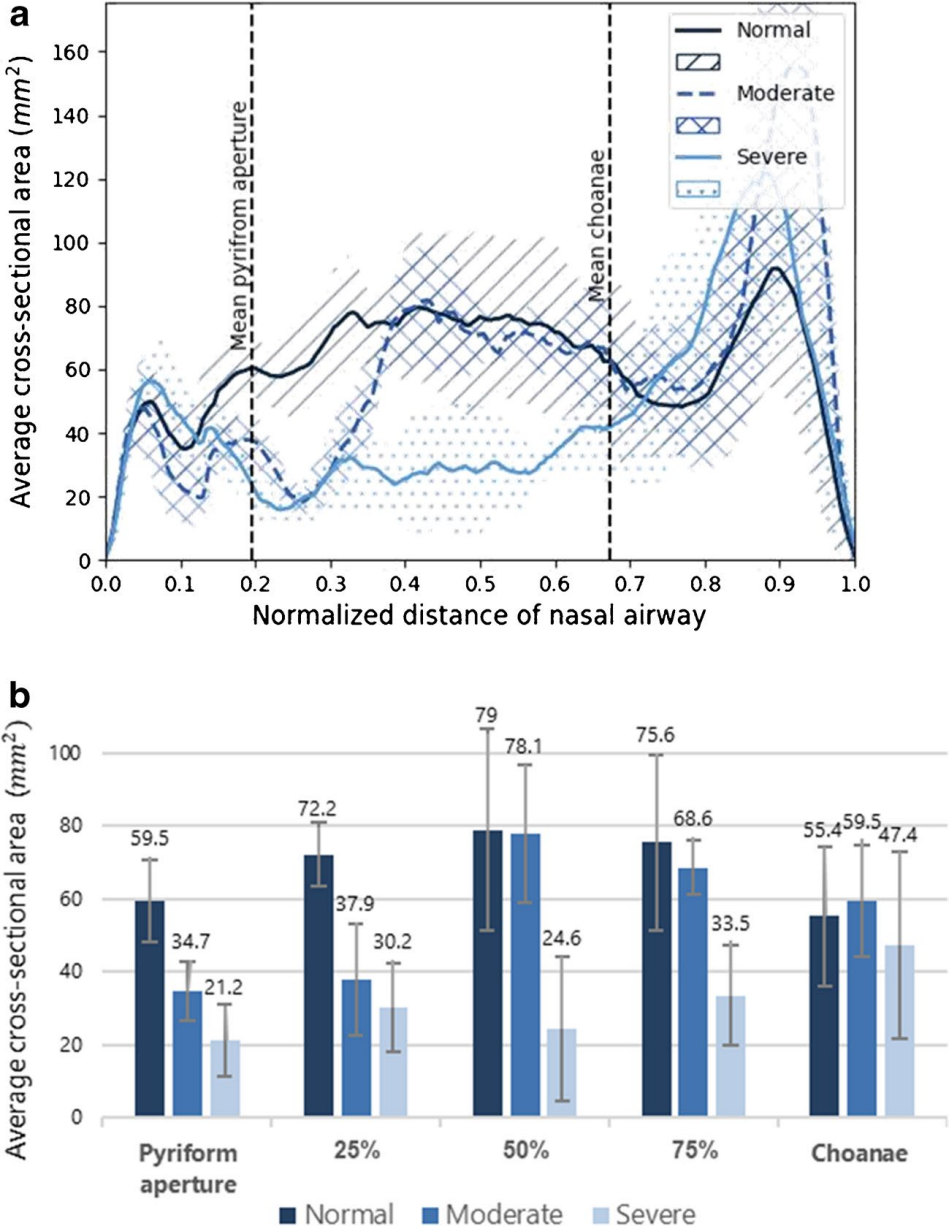
Comparison of the average cross-sectional area along the nasal cavity in three groups: normal (*dark blue*), moderate stenosis (*blue*), and severe stenosis (*light blue*). **a** Graph showing the combined cross-sectional area of both nasal cavities at each axial slice, averaged across subjects in each group. The approximate locations of the pyriform aperture and choanae are marked with *dashed lines*. **b** Group-wise comparison of cross-sectional area at representative locations in the mid-nasal cavity, demonstrating reduced airway dimensions in the severe group compared to the moderate and normal groups

The cross-sectional area comparison graph is depicted in Fig. 5. It should be noted that moderate cases exhibit stenosis in the anterior third of the mid-nasal cavity, whereas severe cases demonstrate narrowing extending from the pyriform aperture to the entire mid-nasal cavity. However, this narrowing becomes less significant before reaching the choanae region. These observations are substantiated by the statistical analysis of the pyriform aperture, 25%, 50%, 75%, and choanae regions, as presented in Fig. 5, with corresponding *P*-values detailed in Table 3.

**Table 3:** The statistical *P*-value of the cross-sectional area in the different locations along the mid-nasal cavity in normal, moderate, and severe cases

Region	Stenosis vs. normal	Moderate vs. normal	Severe vs. normal	Severe vs. moderate
Pyriform aperture	**<0.01**	**<0.01**	**<0.01**	**0.03**
25%	**<0.01**	**<0.01**	** <0.01**	0.24
50%	**0.02**	0.52	**<0.01**	**0.02**
75%	**0.01**	0.47	**<0.01**	**<0.01**
Choanae	0.33	0.66	0.19	0.13

*P*-values below 0.05 are shown in bold to indicate statistical significance

As outlined in Table 3, in moderate cases, stenosis is confined to the pyriform aperture and 25% regions. In contrast, severe cases manifest stenosis in the pyriform aperture, 25%, 50%, and 75% regions, with no statistical significance observed in the choanae. In addition, the cross-sectional area at the pyriform aperture effectively distinguishes between moderate and severe cases, unlike the pyriform aperture width, which is not a reliable gauge of the obstruction severity. Nonetheless, the most conspicuous difference between these two stenosis subgroups is evident in the posterior regions of the mid-nasal cavity, specifically at the 50% and 75% positions, as notably depicted in Fig. 5.

To further evaluate the added value of 3-D measurements over traditional 2-D methods, we manually measured the transverse bone width at the mid-nasal in all cases. The width between the nasal lateral walls was measured halfway between the pyriform aperture and the choana. The measurement was taken perpendicular to the midline plane, parallel to the palatine process of the maxillary bone. This region was selected because the 3-D based cross-sectional area analysis revealed significant differences between the severe and moderate groups in this zone (as shown in Fig. 5). The manual 2-D measurements were performed by Y.S. independently of the segmentation algorithm. The results showed that the average mid-nasal bone width was 9.98 ± 1.61 mm in the severe group, 11.2 ± 0.68 mm in the moderate group, and 15.33 ± 1.04 mm in the normal control group. Statistical comparison showed no significant difference between the severe and moderate groups (*P*=0.20), whereas both the severe and moderate groups differed significantly from the normal group (*P* < 0.01). These results suggest that while manual mid-nasal measurements may detect overall pathological narrowing, they may not adequately distinguish between severity levels within affected cases.

## Discussion

Our study utilized an automated algorithm to extract objective information about the 3-D nasal airway geometry from CT images, revealing significant differences between normal, moderate, and severe pyriform aperture stenosis cases. The algorithm segmented nasal airways with high accuracy and aligned well with manual pyriform aperture measurements. It measured the total volume, regional volume, and cross-sectional area along the nasal cavity of healthy newborns, providing a comparative baseline and a reference for research on airway dimensions development. Key findings include significantly smaller mid-nasal volumes in stenosis cases compared to normal conditions, with significant narrowing extending through the anterior third in moderate cases and up to 75% of the mid-nasal channel in severe cases. The cross-sectional area at 75% of the mid-nasal cavity length emerged as the most significant factor distinguishing moderate from severe cases. To our knowledge, no previous study has examined the difference between surgically and non-surgically treated pyriform aperture stenosis cases from a 3-D perspective or in the posterior parts of the mid-nasal airway.

These findings address critical challenges in diagnosing and treating pyriform aperture stenosis. Currently, while CT scans are used to confirm stenosis diagnosis by measuring pyriform aperture width, treatment decisions are primarily guided by clinical response to conservative management [7, 9, 10]. Some researchers have attempted to predict surgical necessity using pyriform aperture width thresholds [4, 8], but this approach has shown limitations [7, 9, 10] as it provides only a partial image of the stenosis. This careful clinical approach minimizes unnecessary surgeries, though it may not fully utilize the detailed anatomical information available in CT scans that could potentially provide additional insights into symptom severity, support treatment decisions, and optimize surgical planning when intervention becomes necessary. Studies that examined also posterior parts of the nasal channel [11–13] were limited to one-dimensional distance measurements on manually selected two-dimensional cross-sections and did not differentiate between surgical and conservative cases. In our study, we also tested whether manual 2-D measurements at the pyriform aperture or mid-nasal level could distinguish between moderate and severe cases, but no significant difference was found. This highlights the added diagnostic value of full 3-D analysis in assessing the extent and distribution of nasal narrowing in pyriform aperture stenosis. While it is theoretically possible to perform manual measurements at multiple locations along the nasal passage, this process is labor-intensive, time-consuming, and less reproducible.

In contrast, our automated method extracts 3-D data from CT scans, and it offers a comprehensive, objective, and rapid evaluation of the entire nasal passage. It identifies stenosis regions and potentially enables virtual surgical planning and airflow dynamics simulation [17]. Importantly, while no significant difference was found in the transverse dimension of the pyriform aperture between moderate and severe cases, a clear difference was observed in cross-sectional areas at the mid-nasal region. This may have a substantial impact on nasal airflow resistance, as cross-sectional areas directly affect fluid dynamics parameters such as inspiratory flow rate and airway resistance [15]. Considering both anterior and posterior measurements provides additional insights and may help explain why some cases respond differently to conservative treatment, highlighting the relationship between anatomical changes and clinical outcomes. In addition, our results revealed that stenosis cases exhibited approximately one-third larger nasopharynx volumes. Interestingly, this finding might reflect a compensatory mechanism to counteract anterior narrowing and support airflow.

The primary limitations of this study include its small sample size, due to the rarity of pyriform aperture stenosis and ethical considerations regarding radiation exposure in newborns. Future multi-center studies with larger cohorts are needed to validate our findings and establish threshold indicators for surgical intervention based on automatic 3-D measurements. An additional limitation concerns the sensitivity of the automatic algorithm to scanning conditions and image quality. Motion artifacts can affect segmentation accuracy [26], and different segmentation methods may alter measurements [27, 28]. While these factors are unlikely to significantly impact the observed dimensional differences between groups, documenting specific segmentation methods in future research is crucial for meaningful comparisons [28].

Despite these limitations, our study demonstrates the value of automated three-dimensional analysis in improving both surgical decision-making and planning in pyriform aperture stenosis. This precision has the potential to better identify cases where conservative treatment might fail and guide the surgical approach by providing detailed mapping of the stenosis patterns. While further validation is needed in larger cohorts, the combination of automated processing and comprehensive analysis shows promise for optimizing both treatment decisions and surgical outcomes in pyriform aperture stenosis patients. Moreover, while demonstrated here specifically for pyriform aperture stenosis, this automated approach could potentially be adapted for analyzing other types of airway stenoses and might contribute to establishing standardized measurements of normal airway dimensions in children, enabling automated screening for anatomical abnormalities.

## Conclusion

This study presents a newly developed software tool for automated three-dimensional analysis of the nasal airway in newborns. The extracted parameters show potential for assessing pyriform aperture stenosis severity and supporting both the decision to operate and the planning of surgical intervention, although further validation in a larger multi-center cohort is required.

## Supplementary Information

Below is the link to the electronic supplementary material.

Supplementary file1 (DOCX 39 KB)

Supplementary file2 (DOCX 640 KB)

## Data Availability

The code developed in this research, including example implementations, can be accessed at https://github.com/yohod/NeonateNasalAirwayEvaluator.git. Due to privacy regulations, clinical imaging data is not publicly available, but reasonable access requests will be considered subject to institutional approval and data-sharing agreements.
