# Supplementary material for: Automated three-dimensional computed tomography analysis for surgical decisions in congenital nasal pyriform aperture stenosis
Source: Pediatr Radiol. 2025 Jun 24;55(8):1702–12. doi: 10.1007/s00247-025-06282-7 (PMC12321926; doi:10.1007/s00247-025-06282-7)
Supplement: Supplementary file 1 — Supplementary file1 (DOCX 39 KB) [file 247_2025_6282_MOESM1_ESM.docx]

**Automated three-dimensional computed tomography analysis for surgical decisions in congenital nasal pyriform aperture stenosis**

Supplemental tables

**Table S1.** Cases dataset**s -** demographics and clinical information

**Table S2.** Measurements of the pyriform aperture width

**Table S3.** Measurements of the nasal dimension

**Table S1A.** Pyriform aperture stenosis cases dataset - demographics and clinical information

| Treatment | Other abnormalities | Single median central incisor | Pyriform aperture width (mm) | Weight  (kg) | Age  (days) | Gestational age  (weeks + days) | Sex | Case num |
| --- | --- | --- | --- | --- | --- | --- | --- | --- |
| Stents then turbinate reduction | Dermoid cyst,  ventricular septal defect, ectopic kidney, hypoxic ischemic encephalopathy, peripheral pulmonary stenosis | No | 6.7 | 2.1 | 2 | 36 + 2 | Male | **P01** |
| Sublabial approach + stents+  turbinate reduction |  | No | 3.5 | 3.31 | 21 | 42 | Male | **P02** |
| Sublabial approach + stents |  | No | 5.5 | 3.67 | 32 | 40+2 | Male | **P03** |
| Sublabial approach +ּּ lt choanal atresia | Patent Foramen ovale,  atrial septal defect | Yes | 4.6 | 3.3 | 12 | 39+3 | Male | **P04** |
| Sublabial approach + stents |  | Yes | 3.4 | 2.89 | 2 | 40 | Female | **P05** |
| Turbinate reduction + stents | Mild laryngomalacia | No | 4.3 | 3.94 | 8 | 41+1 | Male | **P06** |
| Conservative | Dysmorphism, facial asymmetry, congenital deformity of hand | Yes | 5.1 | 3.0 | 11 | 39+6 | Female | **P07** |
| Conservative |  | No | 4.7 | 3.53 | 4 | 40 | Female | **P08** |
| Conservative |  | No | 5.7 | 3.64 | 3 | 41+6 | Male | **P09** |
| Conservative |  | No | 4.4 | 3.23 | 12 | 38+2 | Male | **P10** |

**Table S1B.** Control cases dataset - demographics and clinical information

| Reason for computed tomography | Pyriform aperture width (mm) | Weight  (kg) | Age  (days) | Gestational age  (weeks + days) | Sex | Case num |
| --- | --- | --- | --- | --- | --- | --- |
| Brain hemorrhage | 12.4 | 3.76 | 34 | 40+1 | Female | **P11** |
| Trauma | 10.6 | 2.71 | 0 | 39 | Female | **P12** |
| Subgaleal  Hematoma | 11.9 | 3.2 | 1 | 40+1 | Male | **P13** |
| Facial edema | 13.2 | 2.74 | 1 | 40+1 | Male | **P14** |
| Trauma | 10.3 | 2.59 | 1 | 39+6 | Female | **P15** |
| Suspected subdural hematoma | 12.1 | 3.88 | 2 | 40 | Male | **P16** |
| Suspected craniosynostosis | 9.5 | 3.19 | 3 | 40 | Male | **P17** |
| Racoon eyes | 12.2 | 3.73 | 6 | 39 | Female | **P18** |
| Trauma | 11 | 3.04 | 4 | 40 | Female | **P19** |
| Trauma | 11.2 | 2.57 | 19 | 38 | Female | **P20** |
| Trauma | 10.7 | 2.66 | 35 | 40+6 | Female | **P21** |
| Trauma | 12.4 | 3.32 | 15 | 39+5 | Male | **P22** |

**Table S2.** Measurements of the pyriform aperture width

| **Automatic width (mm)** | **Manual width (mm)** | **Group** | **Case number** | **Automatic width (mm)** | **Manual width (mm)** | **Group** | **Case number** |
| --- | --- | --- | --- | --- | --- | --- | --- |
| 11.36 | 10.6 | Normal | **P12** | 7.15 | 6.7 | Severe | **P01** |
| 12.21 | 11.9 | Normal | **P13** | 5.16 | 5.5 | Severe | **P02** |
| 11.13 | 13.2 | Normal | **P14** | 3.4 | 4.6 | Severe | **P03** |
| 11.49 | 10.3 | Normal | **P15** | 3.36 | 3.5 | Severe | **P04** |
| 12.3 | 12.1 | Normal | **P16** | 3.18 | 3.4 | Severe | **P05** |
| 11.04 | 9.5 | Normal | **P17** | 4.39 | 4.3 | Severe | **P06** |
| 12.75 | 12.2 | Normal | **P18** | 4.44 | 4.7 | Moderate | **P07** |
| 11.16 | 11 | Normal | **P19** | 4.22 | 5.1 | Moderate | **P08** |
| 11.25 | 11.2 | Normal | **P20** | 5.86 | 5.7 | Moderate | **P09** |
| 11.16 | 10.7 | Normal | **P21** | 3.47 | 4.4 | Moderate | **P10** |
| 12.66 | 12.4 | Normal | **P22** | 13.48 | 12.4 | Normal | **P11** |

**Table S3.** Automatic measurements of the nasal dimensions

| Case number | Total  volume  ($\boldsymbol{c}\boldsymbol{m}^{\boldsymbol{3}}$) | Nares  volume  ($\boldsymbol{c}\boldsymbol{m}^{\boldsymbol{3}}$) | Mid-nasal  volume  ($\boldsymbol{c}\boldsymbol{m}^{\boldsymbol{3}}$) | Nasopharynx  volume  ($\boldsymbol{c}\boldsymbol{m}^{\boldsymbol{3}}$) | surface area  ($\boldsymbol{c}\boldsymbol{m}^{\boldsymbol{2}}$) | Cross-sectional area - pyriform aperture  (mm^2^) | Cross-sectional area 25%  (mm^2^) | Cross-sectional area 50%  (mm^2^) | Cross-sectional area 75%  (mm^2^) | Cross-sectional area choanae (mm^2^) |
| --- | --- | --- | --- | --- | --- | --- | --- | --- | --- | --- |
| P01 | 1.66 | 0.36 | 0.64 | 0.66 | 23.5 | 15.1 | 37.2 | 11.6 | 34.6 | 36.4 |
| P02 | 2.69 | 0.26 | 0.66 | 1.77 | 35.2 | 17.8 | 21.5 | 14.1 | 26.3 | 35.6 |
| P03 | 4.14 | 0.5 | 1.57 | 2.07 | 48.2 | 36.4 | 47.8 | 67.6 | 56.7 | 55.9 |
| P04 | 2.42 | 0.44 | 0.47 | 1.52 | 30.1 | 33.4 | 23.4 | 14.1 | 10.7 | 29.3 |
| P05 | 1.63 | 0.54 | 0.39 | 0.71 | 18.0 | 9.2 | 12.6 | 25.8 | 35.8 | 26.3 |
| P06 | 3.17 | 0.44 | 1.05 | 1.69 | 33.0 | 15.6 | 38.9 | 14.4 | 36.6 | 101.2 |
| P07 | 2.87 | 0.3 | 1.46 | 1.1 | 48.9 | 42.5 | 47.6 | 60.4 | 57.8 | 41.3 |
| P08 | 3.45 | 0.25 | 1.2 | 2.01 | 44.1 | 21.9 | 23.8 | 60.8 | 70.4 | 69.5 |
| P09 | 4.66 | 0.54 | 1.46 | 2.66 | 56.5 | 40.8 | 22.8 | 105.5 | 78.7 | 78.9 |
| P10 | 2.62 | 0.34 | 1.69 | 0.59 | 45.1 | 33.5 | 57.6 | 85.8 | 67.4 | 48.2 |
| P11 | 4.9 | 0.58 | 2.99 | 1.33 | 68.1 | 79.3 | 89.8 | 79.4 | 86.2 | 63.2 |
| P12 | 3.13 | 0.40 | 1.37 | 1.35 | 35.6 | 61.9 | 67.2 | 30.8 | 44.3 | 57.7 |
| P13 | 3.42 | 0.42 | 1.61 | 1.38 | 42.5 | 48.1 | 61.4 | 54.8 | 65.8 | 55.4 |
| P14 | 3.52 | 0.34 | 2.26 | 0.92 | 44.9 | 55.7 | 78.9 | 78.0 | 70.1 | 45.3 |
| P15 | 3.28 | 0.30 | 2.01 | 0.96 | 43.9 | 49.9 | 69.1 | 78.0 | 78.2 | 34.6 |
| P16 | 3.27 | 0.44 | 1.5 | 1.33 | 45.9 | 49.8 | 64.4 | 62.7 | 75.9 | 80.2 |
| P17 | 2.58 | 0.3 | 1.78 | 0.50 | 39.7 | 58.3 | 70.0 | 63.2 | 61.9 | 24.8 |
| P18 | 4.33 | 0.47 | 2.84 | 1.01 | 53.6 | 82.5 | 86.0 | 149.7 | 143.5 | 92.2 |
| P19 | 3.16 | 0.54 | 1.71 | 0.91 | 46.7 | 50.3 | 67.0 | 75.9 | 57.2 | 43.5 |
| P20 | 3.69 | 0.37 | 1.92 | 1.4 | 51.5 | 52.6 | 62.4 | 96.4 | 94.6 | 71.8 |
| P21 | 3.35 | 0.46 | 2.13 | 0.76 | 49.6 | 71.3 | 74.4 | 99.5 | 68.1 | 61.1 |
| P22 | 3.25 | 0.45 | 2.24 | 0.56 | 47.8 | 54.5 | 76.0 | 79.7 | 61.8 | 35.2 |
