# Supplementary material for: Automated three-dimensional computed tomography analysis for surgical decisions in congenital nasal pyriform aperture stenosis
Source: Pediatr Radiol. 2025 Jun 24;55(8):1702–12. doi: 10.1007/s00247-025-06282-7 (PMC12321926; doi:10.1007/s00247-025-06282-7)
Supplement: Supplementary file 2 — Supplementary file2 (DOCX 640 KB) [file 247_2025_6282_MOESM2_ESM.docx]

**Automated three-dimensional computed tomography analysis for surgical decisions in congenital nasal pyriform aperture stenosis**

This supplementary material briefly explains the automatic segmentation algorithm for neonatal nasal airways. The full implementation, compatible with Python version 3.8 and above, is available at: <https://github.com/yohod/NeonateNasalAirwayEvaluator>

# Workflow diagram:

#
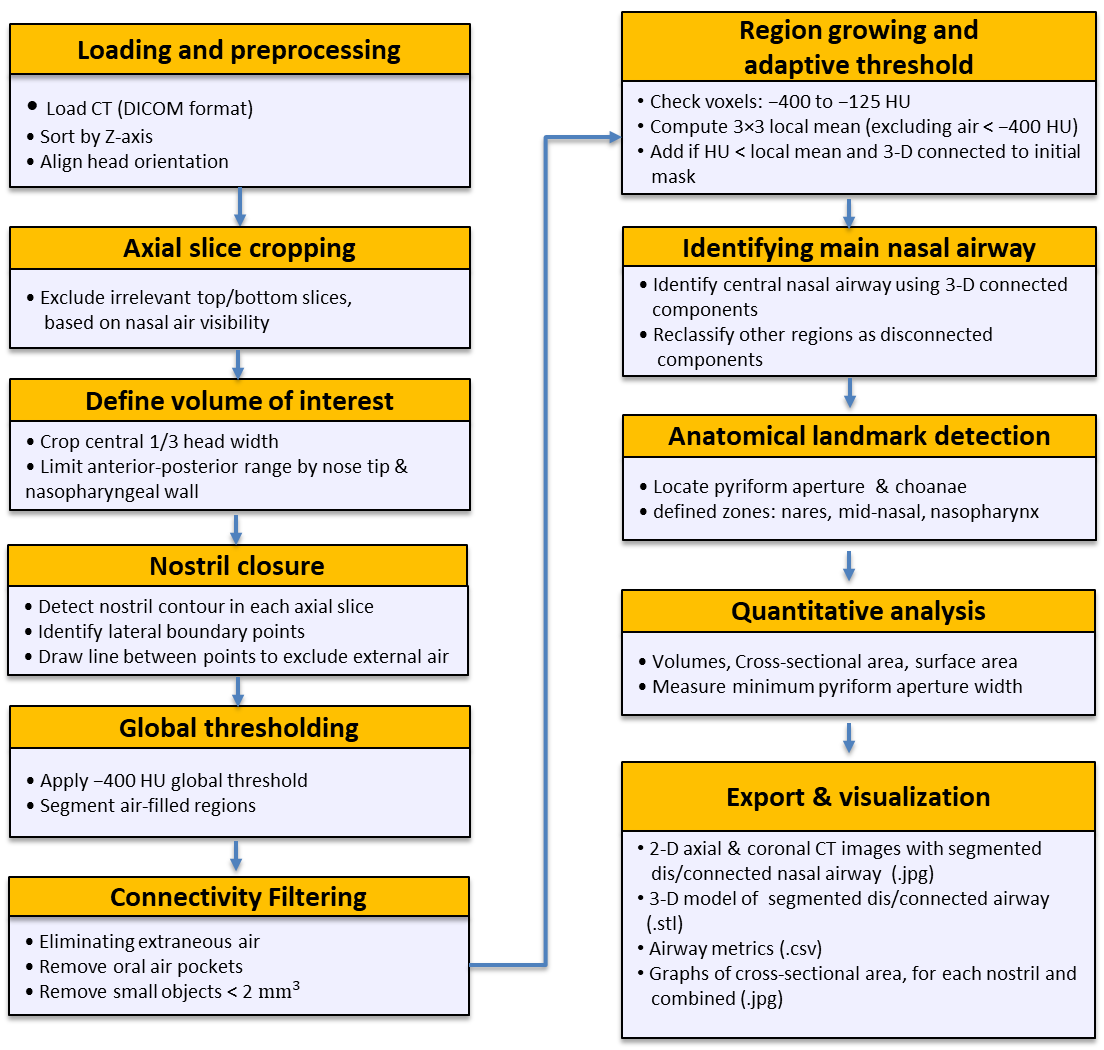


**Figure S1.** The steps of the automatic algorithm. *HU* Hounsfield units, *CT computed tomography, DICOM* digital imaging and communications in medicine*, 3-D* 3-dimensional, *2-D* 2-dimensional

# 1. Loading and preprocessing

The process begins with loading digital imaging and communications in medicine (DICOM) format of computed tomography (CT) scans using the `pydicom` library. Slices are sorted by the Z-coordinate from the `ImagePositionPatient` tag to preserve anatomical order. Since head orientation in neonatal scans can vary, an optional manual reorientation step is proposed, utilizing an affine transformation matrix applied across all slices. This step is facilitated by a simple graphical user interface (GUI) that displays a single mid-axial slice of the head and prompts the user to approximate the rotation angle iteratively until the desired reorientation is achieved.2. Axial slice cropping

Initially, the algorithm performs a rough cropping of the lateral image boundaries up to the sides of the skull, centering the head in the image by applying a global threshold of -525 Hounsfield unit (HU). Subsequently, it searches within the anterior half of the image, starting from the superior slices, to identify the first occurrences of air voxels corresponding to the nasal cavities. Following this, the algorithm locates the nasal tip, defined as the most anterior point of the nasal structure. It then identifies the nasopharyngeal region and tracks its position and dimensions. The search continues inferiorly until the slice where the nasopharyngeal area is minimal, indicating the transition to the oropharynx. Beyond this point, the pharynx begins to connect with the oral cavity. Empirical thresholds for these decisions were determined through manual inspection of training cases and comparison with anatomical references.

# 2. Volume of interest selection

Unlike traditional 3-dimensional (D) bounding boxes, this algorithm uses axial-based region of interest (ROI) selection. From each selected axial slice, the algorithm crops the lateral sides of the image to retain only the central third of the head width, centered on the nasal tip. This approach is based on neonatal craniofacial proportions and excludes the ear cavities. The volume of interest (VOI) in the anterior-posterior axis is dynamically determined by the nasal tip anteriorly and nasopharyngeal wall posteriorly. This method reduces the inclusion of irrelevant structures and improves segmentation specificity by reducing inclusion of irrelevant structures.

# 3. Nostril closure

To isolate the nasal airway from ambient air, the algorithm performs nostril closure within each axial slice that intersects the nasal region. In each such slice, the external boundary of the nostril is automatically detected based on the intensity contrast and morphological outline. Two anatomical boundary points are identified at the lateral ends of each nostril opening. A virtual straight line is drawn between these two points, effectively sealing off the external air from the internal nasal cavity in that specific slice. This slice-wise method provides an effective approximation of nostril isolation without relying on full 3-D facial segmentation.

# 4. Global thresholding

A global intensity threshold of −400 HU is applied to all voxels within the VOI to segment potential air regions. This value was selected as a compromise that avoids over-inclusion of soft tissue while ensuring most of the nasal and nasopharyngeal airways are retained. This step produces a binary mask capturing air-filled areas, including nasal passages and external air.

# 5. Connectivity filtering

The algorithm eliminates the disconnected external air. In addition, using 3-D connected component analysis is employed to remove oral cavity regions disconnected from the nasopharynx, and to filter out small non-continuous regions (<2 mm^3^) to retain only coherent nasal airway structures, thereby improving anatomical accuracy and segmentation robustness.

# 6. Region growing and adaptive thresholding

After the global segmentation, narrow or stenotic airway regions—often missed due to low contrast—are identified using a localized adaptive thresholding method. Only pixels with intensities between −400 and −125 HU are considered candidates for air, as these fall within the range commonly associated with under-segmented airway regions and are below the soft tissue threshold (typically above −100 HU).

For each voxel, the local threshold is computed based on a 3×3 neighborhood within the same axial slice. The threshold is defined as the average of all surrounding pixel intensities (in a 3×3 window) that are greater than −400 HU (to avoid bias from clear air voxels).

A voxel is classified as air only if two conditions are met: (1) its intensity is below the computed local threshold, and (2) it is 3-D connected to the main airway mask segmented by the −400 HU global threshold. This dual condition ensures anatomical continuity and avoids inclusion of isolated artifacts.

Formally, the local threshold T(i,j) for voxel (i,j) is computed as:

$$T(i,j) = \frac{\Sigma_{i}\Sigma_{j} h(x,y)\cdot P(x,y)}{\Sigma_{i}\Sigma_{j} h\left( x,y \right)}$$

where P(x, y) is the voxel HU value, and:
h(x,y) = 0 if P(x,y) < −400 HU and 1 otherwise.

Only voxels satisfying P(i,j) < T(i,j) and 3-D connectivity to the initial mask are added to the final segmentation.

# 7. Identifying the main nasal airway through 3-dimensional connectivity

The algorithm distinguishes the largest 3-D connected component of the segmented air volume—typically representing the main nasal airway—from disconnected regions using a 3-D connected component analysis with 26-neighbor voxel adjacency. This process can aid in identifying completely blocked regions within the nasal cavity.

# 8. Anatomical landmark identification

Two anatomical landmarks are used to segment the airway into three clinically relevant regions: the pyriform aperture and the choanae. The coronal slice of the pyriform aperture was defined as the most anterior slice where bone could be segmented using a +200 HU threshold at the axial level of the nasal tip, which corresponds to the mid-to-inferior region of the pyriform aperture bones. This relatively low threshold allows the detection of under-ossified structures in neonates The choanae are located by scanning from posterior to anterior and measuring the width of the main connected airway region. The choanae are defined as the first slice where the airway width decreases by ≥1.5× compared to the preceding slice, marking the separation of the nasal passages and the transition into the nasopharynx. These landmarks divide the airway into three clinically relevant zones: (1) nares (nostrils to pyriform aperture), (2) mid-nasal (pyriform aperture to choanae), and (3) nasopharynx (posterior to choanae). Their automated identification enables regional analysis and corresponds with common sites of obstruction in congenital conditions.

# 9. Quantitative analysis


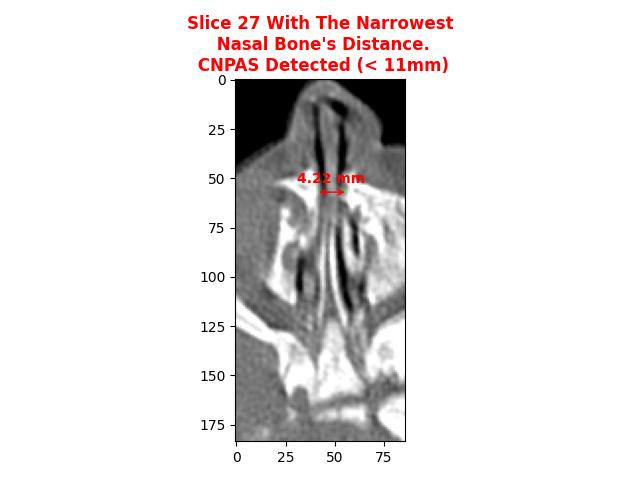
The final segmentation mask is analyzed to extract multiple quantitative parameters. Volume is computed per region by summing voxels and multiplying by voxel size (0.125 mm³). Cross-sectional area is calculated on a per-slice basis using pixel counts. Surface area is estimated via the marching cubes algorithm applied to each regional mask. The minimum pyriform aperture width is measured in the inferior third of the pyriform aperture segment using the shortest medial-lateral distance between the bony boundaries as shown in figure S2. A threshold of <11mm is flagged as abnormal based on literature thresholds for pyriform aperture stenosis.


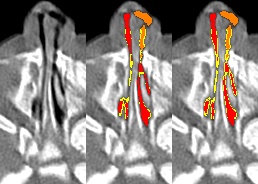

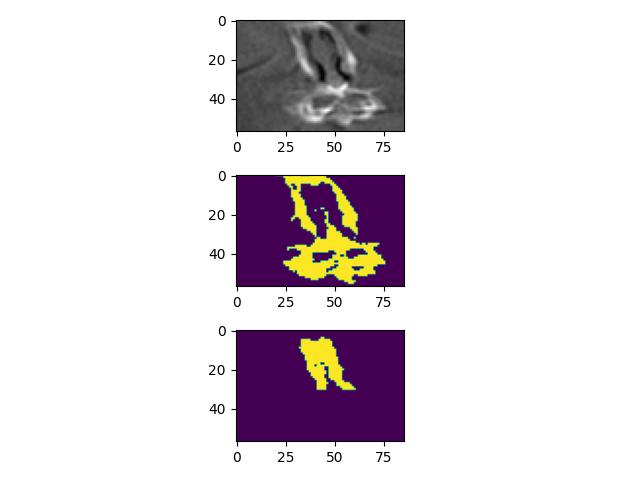


a1

a2


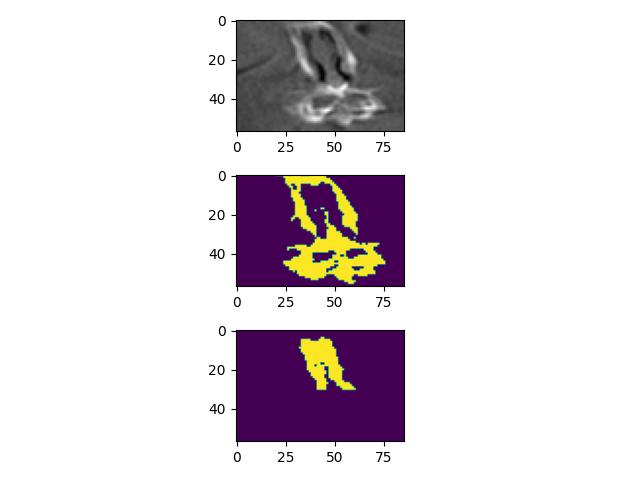


b


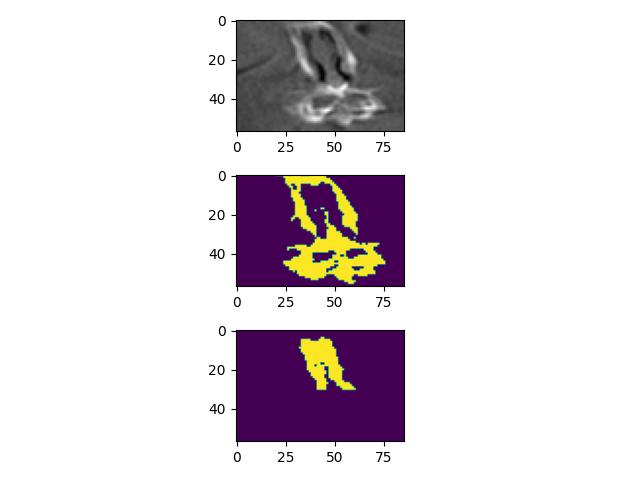


c


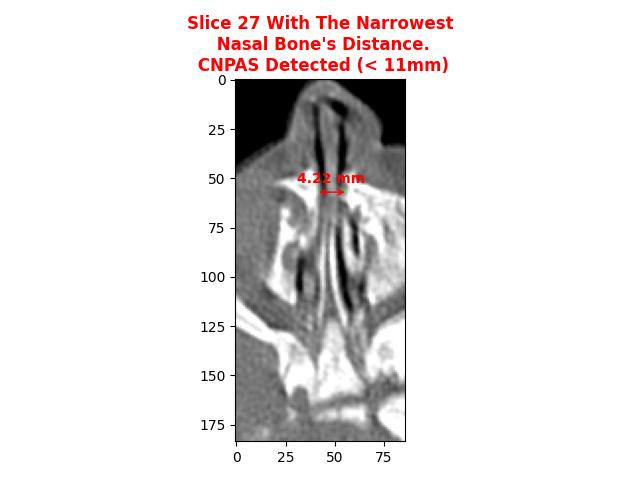


d

**Figure S2.** The steps of the automatic pyriform aperture width measurements. (a) The detected region of interest of the pyriform aperture in the axial (a1) and coronal (a2) planes (b) Bone segmentation using a global threshold of 200 Hounsfield unit (c) Segmenting the internal tissues and the airway. (d) Detecting the axial slice with the narrowest pyriform aperture width and diagnosing the case as a stenosis case

# 11. Export and Visualization

Outputs can include 2-D axial and coronal CT images with the segmentation mask over it (.jpg). CSV files containing all morphological metrics, 3-D surface reconstructions (.STL), Jpg file of the cross-sectional area measurements of each side separately (Figure S3) and cross-sectional area of both sides. Color-coded overlays in 3-D highlight connected vs. disconnected regions, aiding clinical review. The entire process is fully automated and runs in under 15 seconds per scan on standard hardware (Intel i7, 16GB random access memory (RAM)).


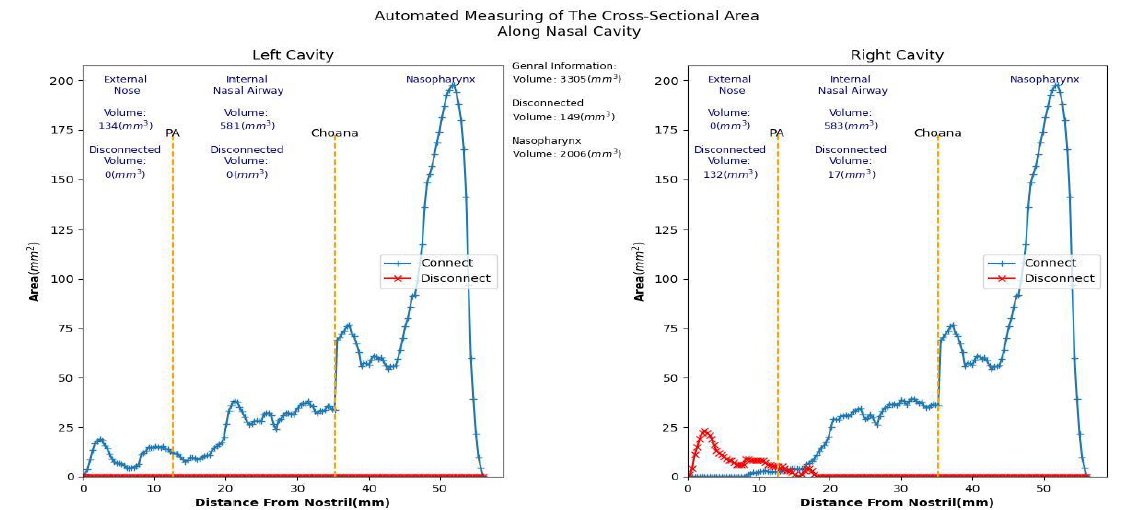


**Figure S3.** Coronal cross-sectional area as a function of distance, with corresponding regional volumes. The blue curve represents connected airway regions, while the red curve denotes anatomically disconnected segments. In this case, a blockage is present in the right anterior nasal canal, as indicated by the disconnection of the right nare from the rest of the airway

**Table S4. Key algorithm parameters** This table summarizes the main parameters used in the segmentation algorithm. All settings were determined empirically and validated on neonatal CT datasets. The algorithm is fully automatic, with a single optional manual step for reorientation when required

| Parameter | Value / range | Purpose | Manually tuned? |
| --- | --- | --- | --- |
| Manual rotation (optional) | graphical user interface | Aligns volume if head orientation is oblique | Yes (optional) |
| Global threshold | -525HU | Initial cropping, define axial VOI slices | No |
| Global HU threshold | −400HU | Initial air voxel segmentation | No |
| Local HU range | −400 to −125HU | Adaptive thresholding for narrow regions | No |
| Region growing connectivity | 26-neighbor (3-D) | Ensures anatomical continuity | No |
| Minimum object size | 2mm^3^ (5-20 voxels) | Removes noise and non-anatomical air pockets | No |
| Nostril closure strategy | Contour-based line per slice | Isolates external air | No |
| Pyriform aperture detection threshold | +200HU, axial slice of nasal tip | Bone segmentation at nasal tip | No |
| Choanae detection | ≥1.5× width change | Detect nasopharynx transition in coronal slices | No |

*3-D* 3-dimensional, *HU* Hounsfield units, *VOI* volume of interest
